# Cathecol and Naphtol Groups in Salphen-Type Schiff Bases for the Preparation of Polynuclear Complexes

**DOI:** 10.3390/ijms21103574

**Published:** 2020-05-18

**Authors:** Samira Gholizadeh Dogaheh, Sara Barbero, Joel Barrientos, Jan Janczak, Janet Soleimannejad, E. Carolina Sañudo

**Affiliations:** 1 School of Chemistry, College of Science, University of Tehran, P.O. Box 14155-6455, Tehran 1417466191, Iran; gholizade_samira@yahoo.com; 2 Secció de Química Inorgànica, Departament de Química Inorgànica i Orgànica, C/MArtí i Franqués 1-11, Universitat de Barcelona, 08028 Barcelona, Spain; sara.barbero.73@gmail.com (S.B.); joel_bcn_9ls@hotmail.com (J.B.); 3 Institute of Low Temperature and Structure Research, Polish Academy of Sciences, P.O. Box 1410, 50-950 Wroclaw, Poland; J.Janczak@intibs.pl; 4 Institut de Nanociència i Nanotecnologia, IN2UB, Universitat de Barcelona, 08028 Barcelona, Spain

**Keywords:** Schiff bases, polynuclear complexes, symmetric ligands, salphen-type ligands, magnetic compounds

## Abstract

In this paper, we show a strategy to modify salphen-type Schiff base ligands with naphtol (SYML1) and pyrocathecol (2,3-dihydroxyphenyl) groups (SYML2), or a combination of both (ASYML). Each of these ligands can be used to obtain polynuclear metal complexes following two different strategies. One relies on using metals that are either too large for the N_2_O_2_ cavity or not fond of coordination number 4 and the other one relies on forcing the polynuclear species by adding functional groups to the hydroxybenzaldehayde in order to have extra coordination sites in the ligand. We report and characterize the mononuclear complexes SYML1-Cu and SYML1-Ce, along with the dinuclear complex SYML1-Fe and the tetranuclear species SYML2-Mn. The asymmetric ligand ASYML routinely hydrolyzes into the symmetric ligands in the reaction mixtures. SYML1-Fe displays a nearly linear Fe-O-Fe bridge with very strong antiferromagnetic coupling between the Fe(III) ions.

## 1. Introduction

There is great interest in the preparation of polynuclear complexes for various application from catalysis to magnetism. The interest in preparing new, better single-molecular magnets and molecular nanomagnets has driven the preparation of ferromagnetically coupled polymetallic [1,2] complexes or heterometallic complexes, such as transition metal-lanthanoid (3d-4f) complexes [3]. Schiff bases can be used for such ultimate goals as ligands that can be prepared in good yields and can be tailored with simple functionalization of either the amine or aldehyde starting materials. In particular, multidentate Schiff base ligands containing imine nitrogen, alcoholic, and/or phenolic oxygen donor atoms can be utilized to synthesize polynuclear complexes with new architectures. Complexes of these ligands with transition and lanthanide ions are one of the most exhaustively studied topics in coordination chemistry [4]. A great example are salen-type ligands with a central ethylenediamine unit [5]. If, instead of ethylenediamine, an aromatic diamine is used, salphen-type ligands are obtained. Salphen-type ligands with an aldehyde functionalized with an OH group are tetradentate. N_2_O_2_ coordinating ligand systems are obtained from the common condensation reaction of one equivalent of a diamine with two equivalents of aldehyde. A symmetric structure ligand is obtained if only one aldehyde is used or asymmetric ligands can be obtained combining two aldehydes [6]. The salphen-type ligands are π-conjugated systems with appropriate photophysical properties. Therefore, these compounds have excellent potential as building blocks in material science among other applications. The choice of a suitable combination of aldehyde and diamine can give rise to extremely luminescent ligands that, combined with paramagnetic metals, can provide multifunctional complexes [7,8]. Asymmetric ligands have special structural and photophysical properties. Thus, in the synthesis of the ligand, the most recent developments have been in the area of asymmetrical ligand synthesis. In fact, a simple method to de-symmetrize the N_2_O_2_ unit can be the prior formation of amine intermediates [9,10,11,12,13].

The great variety of these ligands is based on including aldehydes with fluorescent groups [14]. Aromatic groups amenable for surface deposition or extra oxygen donors makes them a great choice in order to obtain coordination complexes [15]. Metal complexes of symmetric or asymmetric Schiff base ligands are usually prepared by the reaction of the pre-formed ligand with metal and lead to the formation of metal complexes that can be isolated by precipitation or crystallization.

Polynuclear Schiff base complexes are a relevant topic due to their applications in optoelectronic devices [16,17], high thermodynamic stability [18], magnetic materials [19,20,21], sensing, or catalysis [22,23]. Self-assembly of complicated polynuclear complexes can be achieved in many ways such as using Schiff base ligands that can bridge more than one metal. In this way, the structure of the complex obtained is not easily controlled [7,8,15,24,25,26,27,28]. We propose two strategies to obtain polynuclear complexes in a controlled manner using N_2_O_2_ Schiff bases (salen or salphen type ligands). One relies on using metals that are either too large for the N_2_O_2_ cavity or not fond of coordination number 4 and the other one relies on forcing the polynuclear species by adding functional groups to the hydroxyaldehayde in order to have extra coordination sites in the ligand. Usually, salphen-type ligands have an N_2_O_2_ cavity where a transition metal can be easily accommodated and the chelate effect leads to mononuclear species if the metal is comfortable in a coordination number 4. However, lanthanoids are too large for the cavity and sandwich complexes are obtained. Metals that do not favor the coordination number will tend to have extra ligands to attain coordination number 5 or 6 [6,29].

In recent years, our groups have reported several such symmetric and asymmetric Schiff base ligands and their complexes with interesting photophysical and magnetic properties. The interesting results obtained for those complexes inspired us to continue research on the synthesized and dynamic properties of new metal compounds by using various chelating ligands [6,7,8,21]. In continuation of our earlier studies on Schiff base compounds, in this paper, we exploit the two strategies described above to prepare polynuclear complexes with SYML1 and SYML2. The two symmetric ligands SYML1 and SYML2 are prepared with aldehydes with a naphtol unit for SYML1 and a pyrocathecol unit for SYML2, respectively.

## 2. Results and Discussion

### 2.1. Synthesis

**Scheme 1:**
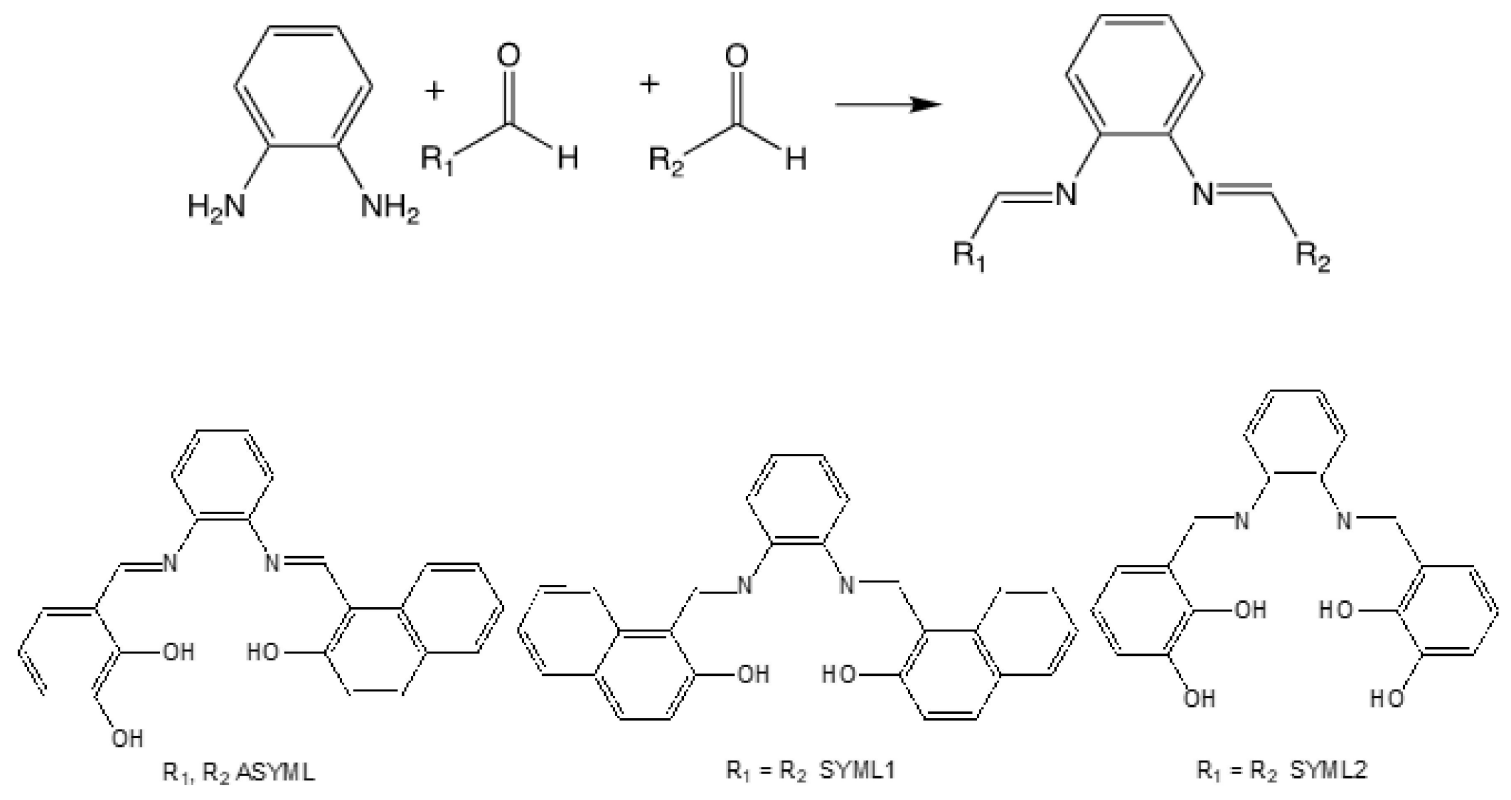
The symmetric ligand SYML1 (N,N0-bis(1-naphthaldiamine)-O-phenylenediamine) was synthesized according to our reported method [6]. This is a straight forward condensation reaction to form an insoluble di-imine that can be easily isolated by filtration in good yield.

The symmetric ligand SYML1 (N,N_0_-bis(1-naphthaldiamine)-O-phenylenediamine) was synthesized according to our reported method [6]. This is a straight forward condensation reaction to form an insoluble di-imine that can be easily isolated by filtration in good yield, as shown in Scheme 1. In our first experience for preparation of asymmetric ligands, we succeeded to synthesize an asymmetric salphen-type ligand with an azo group, AZOL, as reported [6]. This was a two-step process where the intermediate amine-Schiff base was isolated and further reacted in a second condensation reaction to form the asymmetric Schiff base. In our attempts to prepare ASYML, we followed the same two-step reaction shown in Scheme 1. However, in these reaction mixtures of ASYML, SYML1, and SYML2 were obtained. Thus, a one-pot reaction was tested that finally offered the desired product ASYML. In order to obtain a good yield of this pure, bright red solid, the reaction mixture had to be placed in an ice bath. ASYML was characterized by proton NMR and mass spectroscopy, and was found to be pure. The fast formation and very low solubility of SYML1 is likely the reason why this was so. The multiple hydrolysis equilibria in solution are all driven by the fast precipitation of SYML1. The preparation of SYML2 is similar to SYML1, substituting the naphtol group in the aldehyde by a pyrocathecol. It can be filtered from the reaction mixture after the reflux and isolated as pure SYML2 in good yield.

The reactions of SYML1, SYML2, and ASYML with transition metal ions were all tested in similar conditions of room temperature and stirring, or reflux and stirring. Et_3_N was used in all cases as a mild base to deprotonate the OH groups to favor coordination of the ligand to the metals. Following the second strategy described in the introduction, the use of cathecol groups in SYML2 and ASYML should favor the formation of polymetallic species.

The use of the ligand SYML1 with lanthanide ions or transition metals like Fe(III) is an example of the first strategy described in the introduction for the formation of polynuclear complexes. The Lanthanides are too large for the N_2_O_2_ cavity of SYML1 and will tend to form sandwich-type complexes. Furthermore, the charge balance favors a 3 SYML1: 2 Ln stoichiometry for the formed complexes. Furthermore, we and others have observed this fact [6,10,12,13,29,30]. We have continued our study of the reactions of both SYML1 and ASYML with the lanthanide ions. Reaction of SYML1 with Ce(III) salt resulted in an unexpected mononuclear Ce(IV) complex [Ce(SYML1)_2_], SYML1-Ce. The possibility of making cerium complexes in oxidation state +4, even though these complexes might be strong oxidants is shown here. The mononuclear Ce(IV) complex with two ligands is obtained instead of the expected 3 SYML1:2 Ln(III) complex.

In reactions with transition metals, we should distinguish those transition metals like Cu(II) that can form complexes with coordination number 4, and those metals like Fe(III) or Mn(III) that will most likely form complexes with coordination numbers 5 or 6. Reaction of the ligand SYML1 with Cu(II) it is known to afford the expected mononuclear complex [Cu(SYML1)], SYML1-Cu, that we were able to characterize with mass spectroscopy and single-crystal X-ray diffraction. However, reaction with Fe(III) salts yield a di-nuclear complex with an unsupported oxo-bridge between the two Fe(III) ions, [Fe_2_O(SYML1)_2_] SYML1-Fe. In SYML1-Fe, the Fe(III) ions are penta-coordinated.

**Scheme 2:**
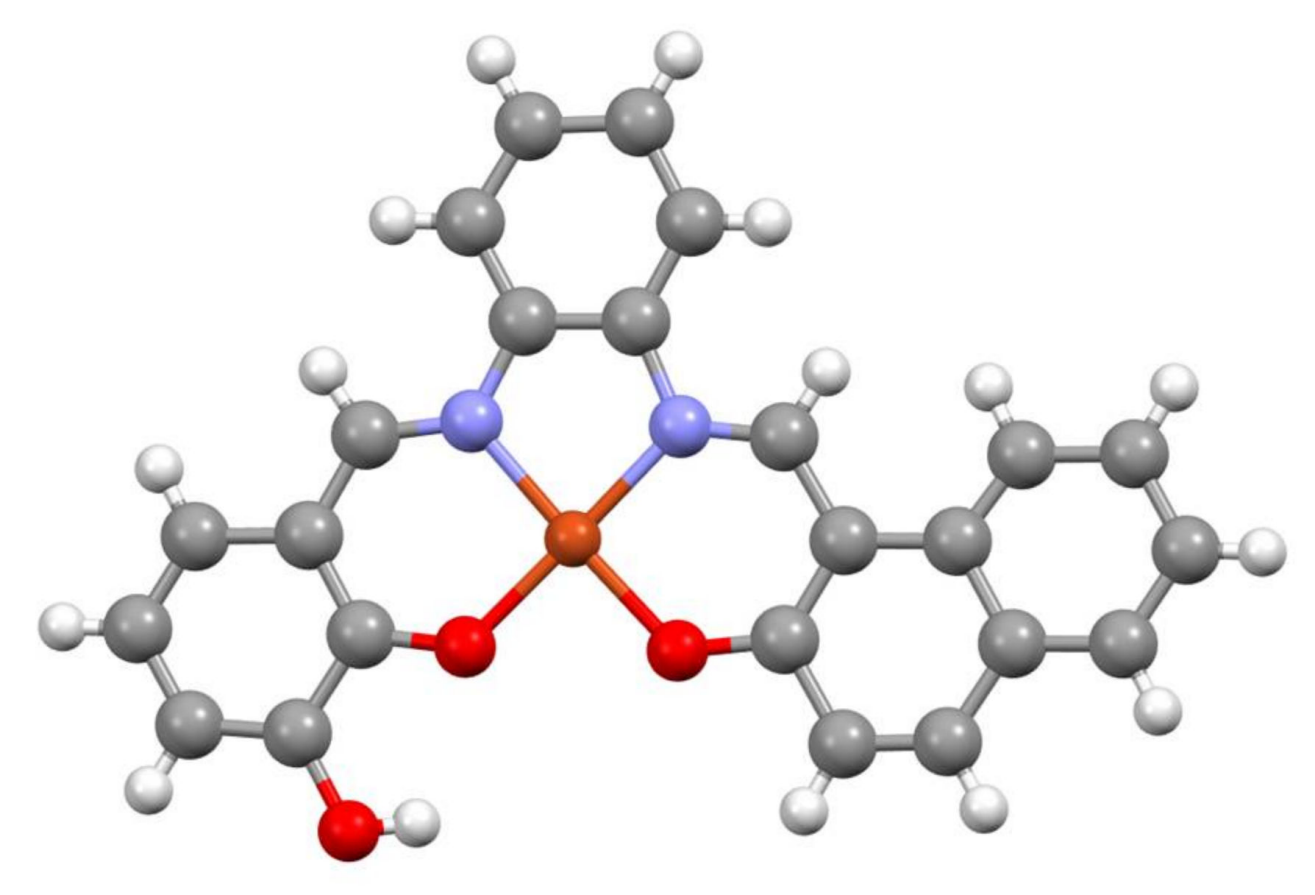
Shows the most probable structure for ASYML-Cu.

Reactions of ASYML with the transition metals resulted in mixtures of products that contained either ASYML or the two symmetric ligands SYML1 and SYML2. Only for Cu(II), we were able to isolate small crystals that were identified by mass spectroscopy and single-crystal X-Ray diffraction as ASYML-Cu with the formula [Cu(ASYMLH)]. The crystal data obtained was of low resolution. Therefore, bond distances and angles cannot be discussed. However, Scheme 2 shows the most probable structure for ASYML-Cu.

From reactions of ASYML with Manganese salts, we learned that it was possible to obtain Mn complexes of SYML2. With this knowledge, we prepared SYML2 and studied the reaction of this ligand with Mn(II) chloride. We were then able to isolate the polynuclear species [Mn_4_(MeO)_2_(SYML2)_2_(MeOH)_4_], otherwise known as SYML2-Mn, in pure, crystalline form from a reaction of hydrated MnCl_2_ with SYML2.

The two strategies proposed to obtain polynuclear complexes in a controlled manner using N_2_O_2_ Schiff bases, mainly the use of metals that are either too large for the N_2_O_2_ cavity or not, fond of coordination number 4, and the addition of functional groups to the hydroxyaldehayde in order to have extra coordination sites in the ligand, have been tested with mixed results. With the ligand SYML1, the use of a lanthanide ion in oxidation state +3 affords the SYML1 to Ln(III) 3:2 complexes previously reported for Tb, Dy, Er, or Yb [6,21]. However, when a similar reaction system is tested with Cerium(III) nitrate instead of the expected triple-decker Ce(III) sandwich complex, we obtained a mononuclear Ce(IV) species. Clearly, Ce(III) oxidized during the reaction and the SYML1 ligand is able to stabilize the Ce(IV) complex. SYML1-Ce is mononuclear since the complex is neutral.

The use of transition metals that can accommodate coordination number four like Cu(II) resulted in the known species SYML1-Cu, which is the expected monomer where the metal has coordination number four. However, the use of Fe(III), which is a transition metal that forms complexes with coordination numbers of at least five, results in the isolation of SYML1-Fe, known as a di-nuclear complex where the Fe(III) ion grabs an oxide to attain coordination number five.

In order to test our second proposed strategy, that is, the addition of functional groups to the basic salphen ligand backbone, we prepared ASYML, where one of the aldehydes bears a pyrocathecol group, as shown in Scheme 1. All our attempts to obtain complexes with the asymmetric ligand ASYML have been unsuccessful due to the equilibria in solution between this asymmetric ligand and the formation of the symmetric counterparts SYML1 and SYML2. We have only been able to observe ASYML-Cu complexes by mass spectroscopy. The peaks Ms/z (M+1H^+^) = 444.06. (M+Na^+^) = 467.06. (2M+H^+^) = 889.10 were observed, which correspond to ASYML-Cu with one proton, ASYML-Cu with one Na+, and 2 [Cu(ASYML)] units with one extra proton. This exhibits that the ASYML complexes can be obtained, but isolation is still elusive. We feel that these data are not enough to give the complex a correct molecular formula, since mass spectra of the polynuclear complexes shows, in all cases, 1 ligand: 1 metal unit due to fragmentation in the conditions of mass spectroscopy and, often, combination of these units. From this reaction, we isolated some crystals, that were too small, and the formula observed by mass spectrometry was confirmed for the Copper-ASYML complex as the mononuclear species [Cu(ASYMLH)], ASYLM-Cu. However, the data were not good and the reproducibility was complicated by the isomerization of the asymmetric ligand in the solution to SYML1 and SYML2. The crystallographic data is shown in Electronic Supplementary Information (ESI) Table S3.

In conclusion, from our experiments, we can determine that it is clear that the equilibria in solution of asymmetric Schiff bases precludes easy isolation of metal complexes of these ligands, and very specific conditions must be met in order to obtain asymmetric Schiff base complexes in pure form. From reaction mixtures of manganese salts and ASYML, we were able to find peaks in mass spectroscopy only from fragments with one symmetric ligand and one manganese ion, which corroborates that isolation of manganese complexes with ASYML was not possible, even though we had observed the contrary for Cu(II). However, the observation of a SYML2 fragment with one manganese ion lead us to the preparation of the symmetric salphen type ligand SYML2 with pyrocathecol groups. Now SYML2 has four oxygen donor atoms and two nitrogen atoms. Reaction of this ligand with hydrated manganese(II) chloride gives the new, mixed-valent tetranuclear complex SYML2-Mn. Upon reaction, the mixture turns dark brown, which indicates the expected oxidation of Mn(II) to Mn(III) for a reaction done open to the atmosphere in the presence of a mild base. The hydroxo groups that are not part of the N_2_O_2_ unit acts as bridges, linking two Mn ions, as shown in Scheme 2, and as described in the crystal structure section. This result shows that it is possible to add further functional groups to the aldehyde in salphen-type ligands in order to obtain polynuclear complexes.

### 2.2. Description of Crystal Structures

**Figure 1:**
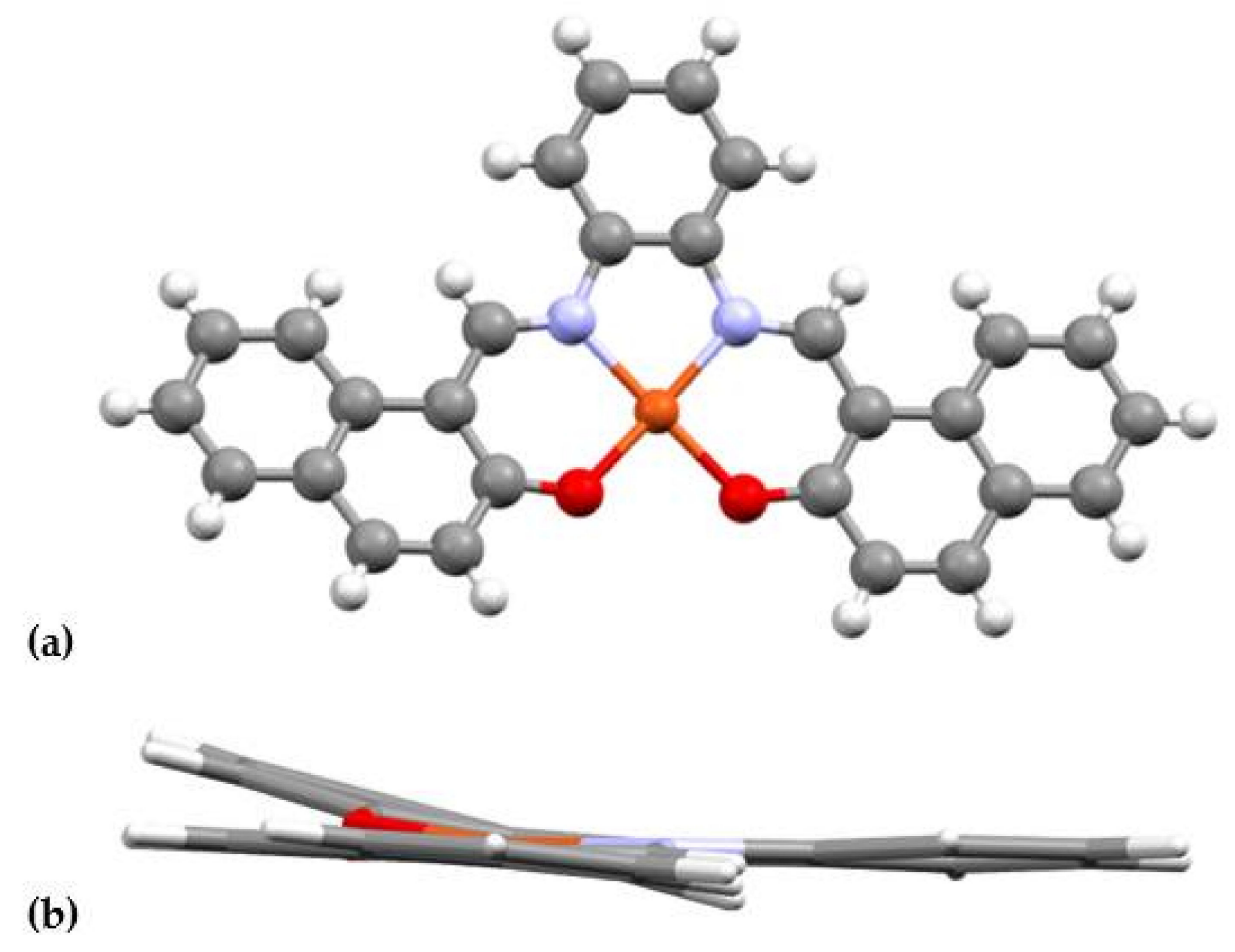
(**a**) Crystal structure of SYML1-Cu. Carbon: gray, hydrogen: white, oxygen: red, nitrogen: blue, Cu(II): orange. (**b**) Side view of SYML1-Cu, showing the distortion from planarity.

Table 1 summarizes crystallographic parameters for the reported crystal structures. ESI Table S3 contains the crystallographic parameters for the low ASYMLH-Cu. Bond valence sum results for all reported species can be found in ESI Tables S1 and S2. SYML1-Cu crystallizes in the monoclinic space group P 2_1_/n. The crystal structure is shown in Figure 1. The Cu(II) is tetra-coordinated in a distorted square planar environment with L-Cu-L angles around Cu(II) of 84.81° and 93.27°. The Cu–O and Cu–N distances are 1.893 Å and 1.932 Å as expected for Cu(II) [31].

**Figure 2:**
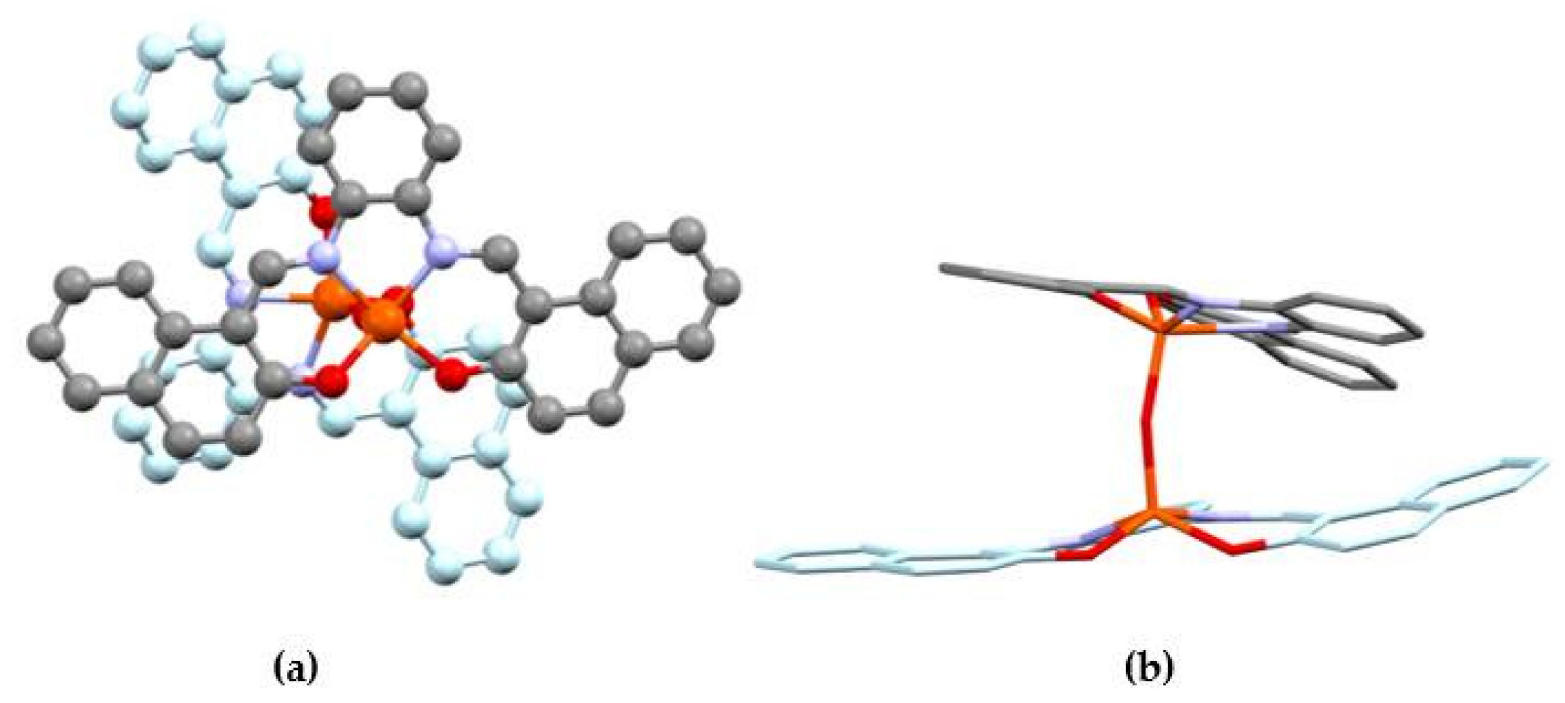
(**a**) Crystal structure of SYML1-Fe, top view. (**b**) Side view of SYML1-Fe. One ligand is shown in light blue and hydrogens are not shown for clarity. Carbon: gray. Oxygen: red. Nitrogen: blue. Iron: orange.

SYML1-Fe crystallizes in the monoclinic space group C 2/c. The crystal structure is shown in Figure 2. It contains two Fe(III) atoms that are penta-coordinated in a distorted square pyramid environment. The base of the pyramid is formed by the N_2_O_2_ cavity of the SYML1 ligand and the apical position is taken by the unsupported oxo bridge. For this bridging ligand, the Fe–O distances are 1.767 Å and the Fe–O–Fe angle is 144.58°. The angle found is relatively common for the [Fe(III)-O-Fe(III)] unit. Several species with nearly linear Fe-O-Fe units have been reported and studied in detail in the bio-inorganic chemistry field [32,33]. Naphthalene groups generate steric repulsion between the two [Fe(SYML)] units on one end of the complex and a stabilizing CH-π interaction on the other side, giving the species the bent structure observed in Figure 2b. The two SYML1 ligands are in a staggered conformation usually observed for sandwich complexes of phthalocyanine [34] or salphen-type ligands [6,14].

**Figure 3:**
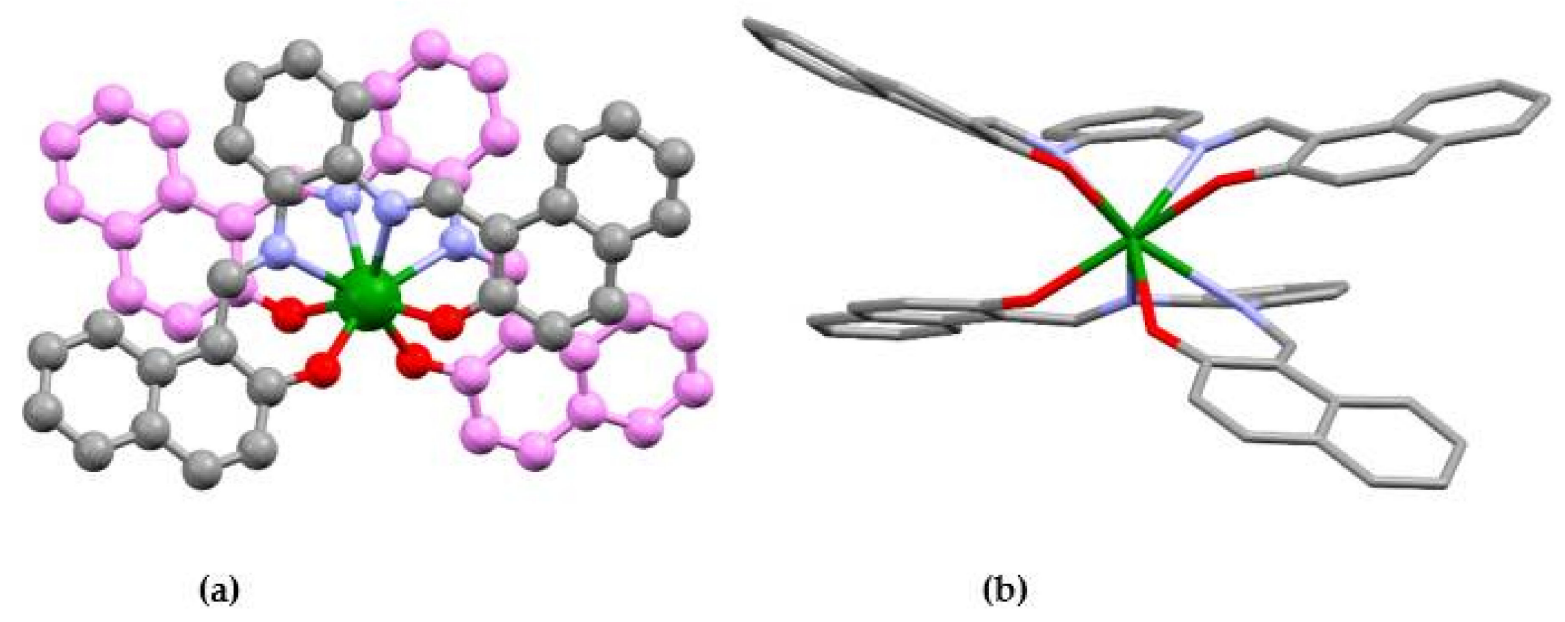
(**a**) Crystal structure of SYML1-Ce. One ligand is shown in light purple and hydrogens are not shown for clarity, top view. (**b**) Side view of SYML1-Ce. Carbon: gray. Oxygen: red. Nitrogen: blue. Cerium: green.

The crystal structure of SYML1-Ce is shown in Figure 3. SYML1-Ce crystallizes in the orthorhombic crystal system of the P2_1_2_1_2 space group. This structure contains one Cerium(IV) sandwiched between two SYML1 ligands. The Ce(IV) ion is eight-coordinated with four N atoms and four O atoms from two N_2_O_2_ SYML1 ligands. The coordination polyhedron can be described as the usual distorted square antiprism observed for Ln(III) ions. Bond valence sum (BVS) can be used to correctly assign the oxidation state of cerium ion in this complex [35]. The Ce(IV)–O and Ce(IV)-N coordination bond lengths are within the expected ranges for cerium ion in this oxidation states and types of ligands [36]. The intramolecular distance Ce–O are in the range of 2.211(3)–2.236(3) Å and the average distance of Ce–O bonds is 2.223 Å and the Ce–N bond lengths are in the range of 2.531(4)–2.581(4) Å is in good agreement with the references.

**Figure 4:**
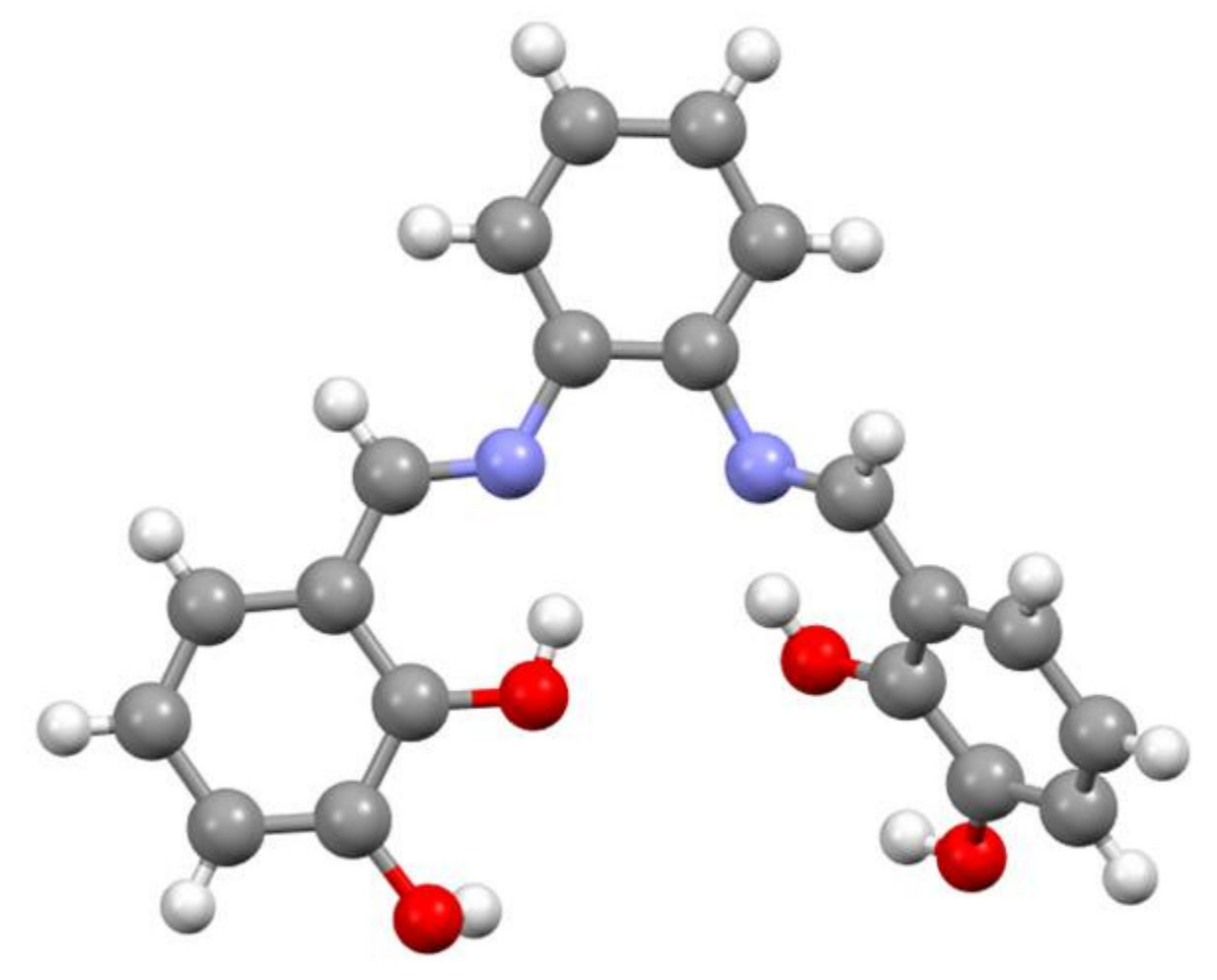
Crystal structure of the symmetric ligand SYML2. Carbon: gray. Hydrogen: white. Oxygen: red. Nitrogen: blue.

SYML2 crystallizes in the Monoclinic space group P21/n. The crystal structure of free ligand SYML2 is shown in Figure 4. The ligands adopt a non-planar configuration due to the steric hindrance of the OH groups. This ligand has been reported before, and our studies corroborate the non-planar solid-state structure [37]. The torsion angle O–N–N–O is 36.12°. This molecule has six donor atoms (4O, 2N). There are two imine groups with a C=N distances of 1.272 Å and 1.287 Å. There are two intramolecular hydrogen bonds between the N and the H of the nearest hydroxyl group with bond distances between the two heavy atoms of 2.529 Å and 2.628 Å.

**Figure 5:**
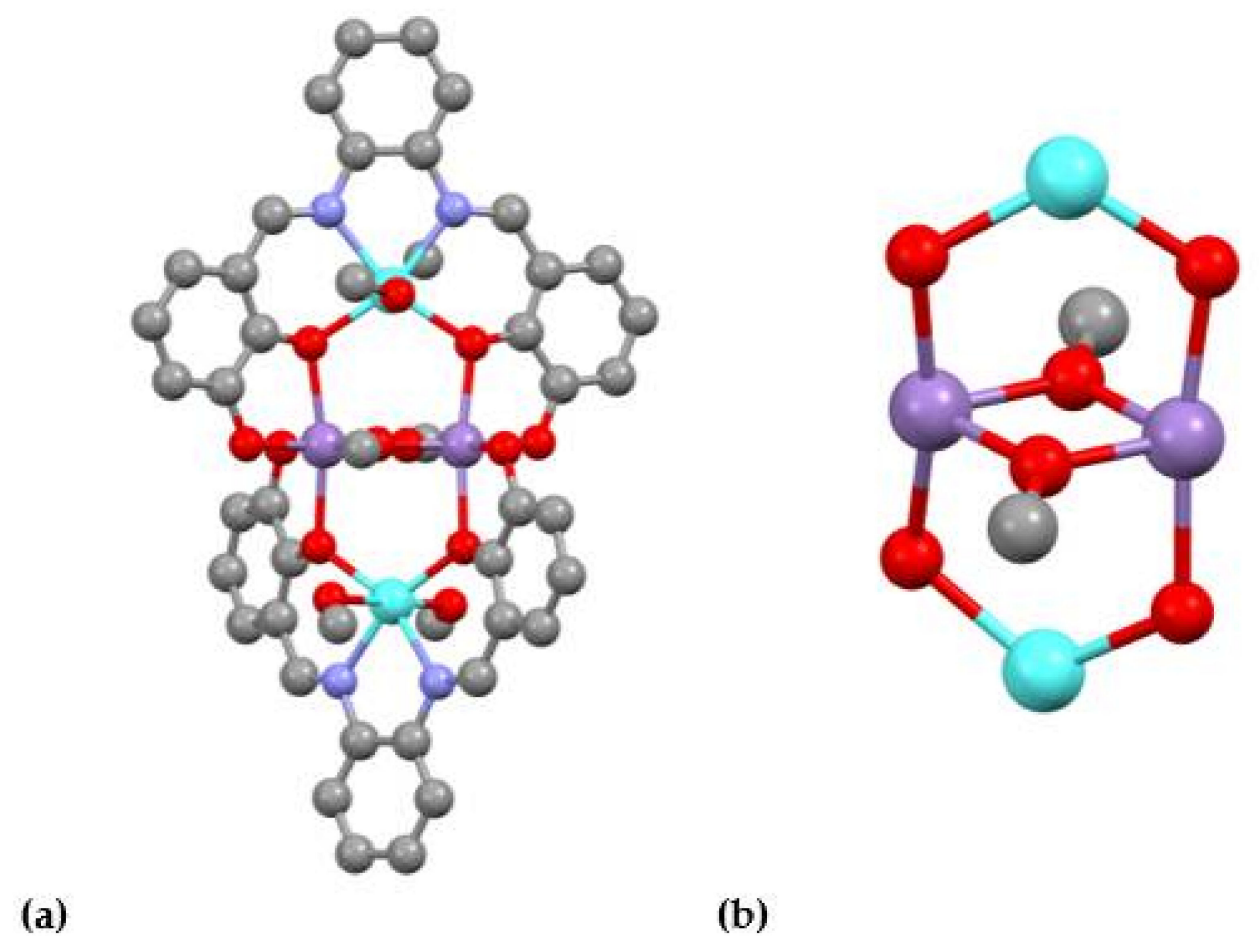
Crystal structure of SYML2-Mn. (**a**) Molecule, without hydrogens for clarity. Carbon: gray. Oxygen: red. Nitrogen: blue. Mn(II): cyan. Mn(III): purple. (**b**) The Mn-oxide core showing the two methoxo bridges.

SYML2–Mn crystallizes in the Triclinic space group P-1. The crystal structure is shown in Figure 5. This type of Mn-O core has been seen before in the literature. However, it is not common [38]. SYML2-Mn is a tetranuclear mixed-valent complex with two Mn(III) and two Mn(II). The two Mn(II) are hexa-coordinated in a distortional octahedral environment with an equatorial chelating SYML2 ligand and two MeOH groups taking the axial positions (Mn(II)-O distances are 2.268 Å and 2.295 Å). The SYML2 ligand is completely deprotonated and the donor atoms are two nitrogen and the two nearest oxygens. The Mn(II)–O distances are between 2.067 Å and 2.077 Å and Mn–N distances are 2.177 Å and 2.187 Å, as expected for Mn in oxidation state +2. The N_2_O_2_ cavity of the ligand forces some distortion from the ideal octahedral geometry with angles around the metals of 76.58° (N–Mn–N) and 113.53° (O–Mn–O) for one of the Mn^II^ and 76.67° (N–Mn–N) and 111.43° (O–Mn–O) for the other one. The two Mn(III) are hexa-coordinated. Each one is coordinated to six oxygen atoms. Four are provided by the SYML2 ligands and by the other two by two bridging methoxy groups. The axial Mn–O (oxygen coordinated to Mn(II)) are 2.165 Å and 2.198 Å, while the equatorial Mn–O are 1.912 Å and 1.922 Å (O from SYML2) and 1.936 Å and 1.945 Å (O from bridging methoxide). The Jahn–Teller distortion in Mn(III) is confirmed by these distances, which resulted in an elongated octahedron. Each SYML2 ligand is not flat. In fact, the torsion angles (O–N–N–O) are 14.77° and 14.50°, but defining an idealized N_2_O_2_Mn plane, the two ligands are rotated 136.5° with respect to each other.

### 2.3. Magnetic Properties

Magnetic susceptibility data were collected for SYML1-Fe, SYML1-Ce, and SYML2-Mn at 300 Oe between 2 to 30 K and at 3000 Oe between 2 to 300 K. Bond valence sum (BVS) studies supported the oxidation state Ce(IV) for cerium in SYML1-Ce. The lack of magnetic moment for this species further confirms this oxidation state.

**Figure 6:**
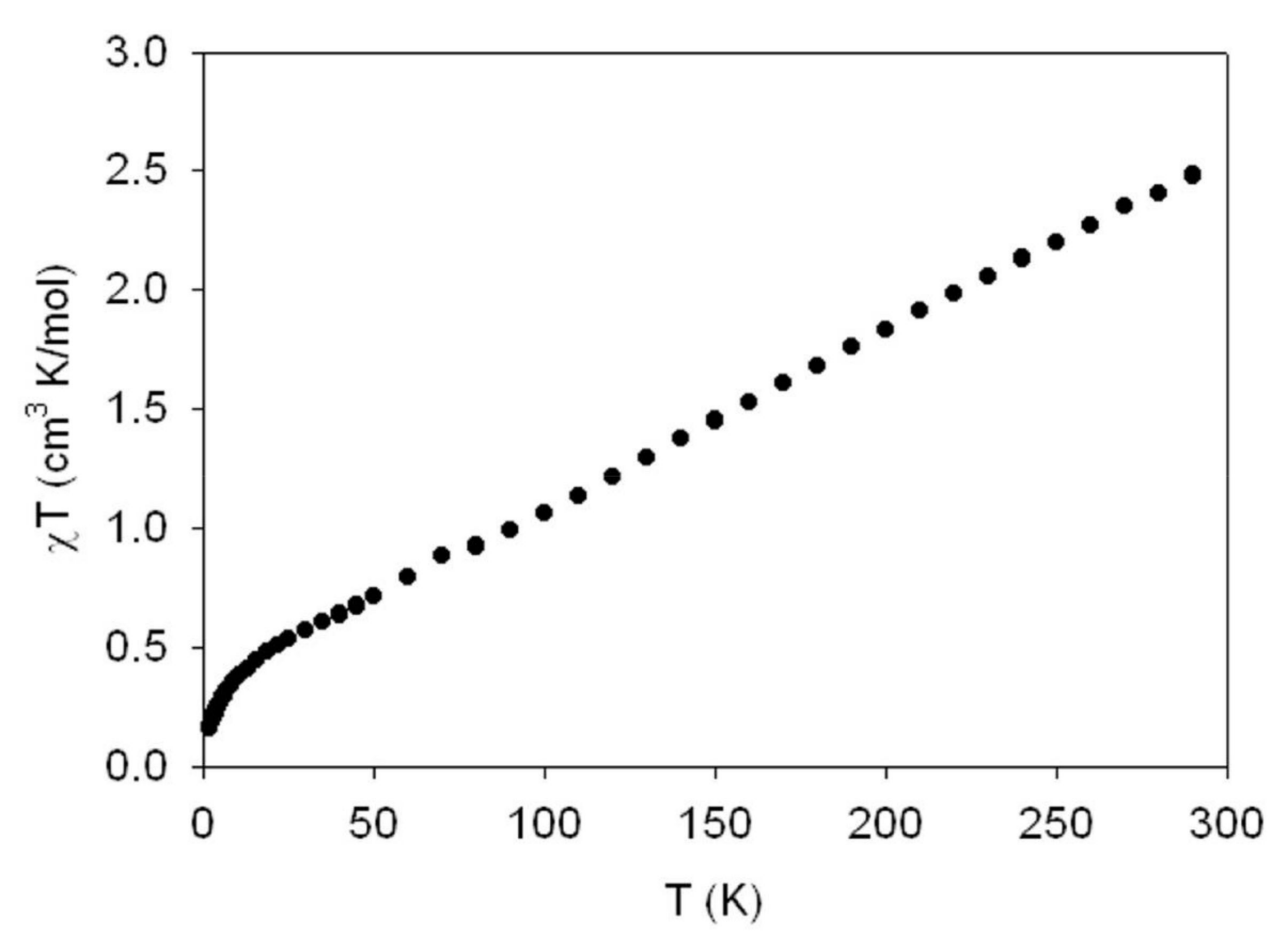
χT vs. T plot for SYML1-Fe measured with an applied dc field of 0.5 T.

The plots of χMT vs T and M vs H for SYML1-Fe is shown in Figure 6. The χT value at 300 K is 2.536 emu K/mol. This value is much lower than the expected value with the calculated value of 8.75 emu K/mol for two free Fe(III) ions. The observed low value reflects a strong antiferromagnetic interaction. The magnetization vs. field data collected at 2 K show values between 0–0.246 μB at 0–50000 Oe, as expected at 2 K for two Fe (III) ions with a strong antiferromagnetic interaction that leads to an S = 0 ground state.

The unconventional, nearly linear, unsupported Fe-O-Fe motif lead us to study the magnetic properties of SYML1-Fe. The magnetic properties of exchange coupled di-nuclear complexes of transition metal ions are known to depend on the particular metal ions, the chemical nature of the bridging ligands, and the bridging geometries. Both bridging angles and bridging ligand-metal distances are of importance. The magnetic properties are given in terms of J and g, which are the parameters of the effective Hamiltonian $\hat{H}=-J{\hat{S}}_{1}\cdot {\hat{S}}_{2}+g\mu_{B}\left({\hat{S}}_{1}+{\hat{S}}_{2}\right)$ (**1**) with *S_1_ = S_2_ =* 52 for Fe(III). There have been several empirical and theoretical attempts to find the relationship between J values of oxo-bridged iron(III) dimers and the Fe—O distances or the Fe—O—Fe angle. A rapid decrease in the absolute value of J is expected upon decreasing the angle from 180°. In 1997, Högni et al. showed that, using the angular overlap model, an expression can be derived that depends on both the mean Fe-O distance r (in Å) and the Fe-O-Fe angle φ, as shown in Equation (1) [33].

(1) $$-J_{model}=1.377\cdot 10^{8}(3.536+2.488\cos\varphi +cos^{2}\varphi )\;x\;\exp\left(-7.909r\right)$$

We used this equation to predict the coupling in our system Fe(III) di-nuclear system SYLM1-Fe. The antiferromagnetic exchange-coupling constant J was calculated for an average Fe-O distance r = 1.767 Å and Fe-O-Fe angle φ = 144.58°, as measured from the crystallographic data. The model yields *J****model*** *=* −255 cm^−1^, which is a strong antiferromagnetic interaction (it is worth to note here that we have used the common convention of positive exchange = ferromagnetic and negative exchange = antiferromagnetic. The opposite convention is used in the Güdel paper). This is in agreement with the values shown in the literature for similar Fe-O-Fe systems (values with different Hamiltonian conventions are between -160 to - 265 cm-1). The strong antiferromagnetic coupling clearly explains the experimental χT vs. T and M vs. H data. With such predicted strong antiferromagnetic coupling, it is not possible to obtain an acceptable fit for the data, since the complex is diamagnetic in most of the temperature interval studied and the data show only Zeeman population of excited states and a small amount of Fe(III) mononuclear impurity in the sample. A fit is shown in Figure S2 in ESI.

**Figure 7:**
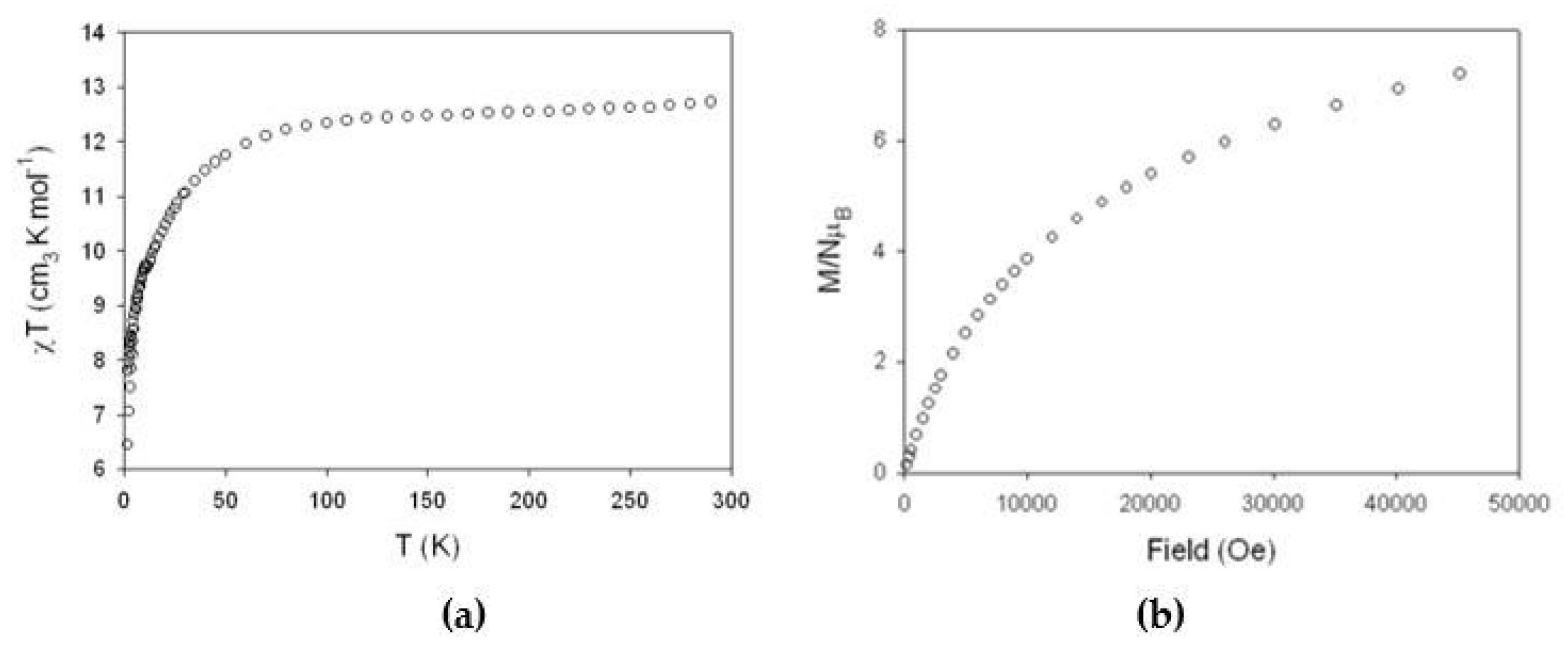
(**a**) χT vs. T plot for SYML2-Mn measured with an applied dc field of 0.5 T. (**b**) Magnetization vs. field plot for SYML2-Mn at 2 K.

The susceptibility data for SYML2-Mn is shown in Figure 7 as χT vs. T and M vs. H plots. At 300 K, the observed χMT value is 12.72 emu K/mol, which is in agreement with the expected value of 12.75 emu K /mol for two Mn(III) (S = 2, g = 2.0) and two Mn(II) (S = 5/2, g = 2). As temperature decreases, the χT product decreases very little until 100 K. Below this temperature, a sharp decrease is observed that indicates antiferromagnetic interactions in the tetranuclear core of SYML2-Mn. The magnetization vs. field plot (see Figure 7) shows that the magnetizations rise continuously without reaching saturation at 5 T. This is consistent with having a large number of excited states very close in energy to the ground state that are populated due to the Zeeman effect. The system seems to be heavily frustrated and the fittings are meaningless, since the ground state has many excited estates close in energy. Thus, there is no clear minima in the error surface for the fitting.

Due to the presence of Mn(III) in the complex SYML2-Mn, ac magnetic susceptibility data were collected. SMM behavior is well known for Mn(III) complexes. Unfortunately, no out-of-phase signal in the ac magnetic susceptibility was observed for SYML2-Mn in the 1 to 1500 Hz and the 0 to 32 Oe dc field range. The complex does not display single molecule magnet (SMM) behavior.

## 3. Materials and Methods

All the chemicals and solvents were purchased from commercial sources and used as received. The symmetric ligand SYML1 was prepared as previously reported [6]. ESI Figure S3 shows thw experimental and calculated mass spectra for SYML1-Cu, ASYMLH-Cu, SYML1-Fe and SYML2-Mn.

SYML2: (3,3′-((1E,1′E)-(1,2-phenylenebis(azaneylylidene))bis(methaneylylidene))-bis(ben-zene-1,2-diol): 0.433 g (4 mmol) of benzene-1,2-diamine were dissolved in 15 mL of absolute EtOH. 1.105 g (8 mmol) of 2,3-dihydroxybenzealdehyde were added to the previous solution. The resulting orange solution was refluxed for 2-3 h at 78 °C. During the reflux, the solution changed to an intense red color. After the reflux, an ice-bath is performed. The red solid obtained was filtered and washed with diethyl ether. Yield: 88%.

IR (ATR, cm^−1^): ^1^H NMR (400 MHz, CDCl_3_): δ 13.47 (s, 1H), δ 9.83 (s, 1H), δ 8.63 (s, 1H), δ 7.38 (dd, J = 5.9, 3.4 Hz, 1H), δ 7.28 (dd, J = 5.9, 3.4 Hz, 1H), δ 7.05 (dd, J = 7.9, 1.5 Hz, 1H), δ 6.97 (dd, J = 7.8, 1.5 Hz, 1H), δ 6.84 (t, J = 7.8 Hz, 1H). LRMS (ESI): (C_20_H_16_N_2_O_4_) MW = 348 g/mol Ms/z (M+1H^+^) = 349.12. UV–Vis: 280 nm, 335 nm, 475 nm. Fluorescence (emission): 400 nm and 460 nm (λ_ex_ = 335nm).

ASYML: (3-((E)-((2-(((E)-(3-hydroxynaphthalen-2-yl)methylene)amino)-phenyl)imino)methyl)benzene-1,2-diol): 0.39 g (3.62 mmol) of benzene-1,2-diamine were dissolved in a minimum quantity of absolute EtOH. Additionally, 0.565 g of 3-hydroxy-2-naphthaldehyde and 0.500 g (3.62 mmol) of 2,3-dihydroxybenzealdehyde were added to the previous solution. The resulting orange solution was refluxed for 1 h at 78 °C. After this time, the color of the solution changed to a more intense orange/red. The solution was cooled in an ice bath. Diethyl ether was added in order to make it precipitate. The red solid obtained was filtered and dried with diethyl ether. Yield: 55%.

IR (ATR, cm^−1^): 1610(s), 1586(m), 1544(m), 1485(m), 1347(m), 1273(s), 1194(m), 1155(w), 962(w), 754(s), 729(s). ^1^H NMR (400 MHz, CDCl_3_) (ESI Figure S4) δ 15.29 (1H), δ 13.16 (s, 1H) δ 9.35 (d, J = 5.0 Hz, 1H), δ 8.68 (s, 1H), δ 8.08 (d, J = 8.5 Hz, 1H), δ 7.80 (d, J = 9.1 Hz, 1H), δ 7.70 (d, J = 7.3 Hz, 1H), δ 7.51 (d, J = 8.1 Hz, 1H), δ 7.48–7.27 (m, 5H), δ 7.10 (d, J = 9.1 Hz, 1H), δ 7.06 (d, J = 7.9 Hz, 1H), δ 7.00 (d, J = 7.7 Hz, 1H), δ 6.85 (t, J = 7.9 Hz, 1H). LRMS (ESI): (C_24_H_18_N_2_O_3_)MW = 382 g/mol Ms/z (M+1H^+^) = 383.14. UV–Vis: 270 nm, 310 nm, 400 nm, 475nm. Fluorescence (emission): 370 nm and 480nm (λ_ex_ = 310nm).

SYML1-Ce: Ce(NO_3_)_3_.6H_2_O (0.434 g, 1 mmol) is dissolved in 20 mL of CH_3_CN. To this stirred solution, 0.416 g (1 mmol) of SYML1 in 50 ml of CH_3_CN was added and heated under reflux. After the addition of Et_3_N (5 mmol), the color changed from orange to brown and a precipitate formed. The resulting solution was stirred and heated at reflux for 5 h. The precipitate was filtered off, washed with hot acetonitrile three times, and then dried with anhydrous diethyl ether. Yield: 74%. Dark orange crystals of SYML1–Ce were obtained after one week by slow evaporation of the solution. IR (ATR, cm^−1^): 1607(m), 1571(m), 1532(m), 1321(m), 1173(w), 1083(w), 975(w), 823(w), 741(s), 505(s), 445(m). A detailed UV.vis study can be found in ESI page 1 and Figure S1.

UV–Vis: λmax (nm) ( ɛ(M^−1^ cm^−1^)): 225 nm, 380 nm and 448 nm in CH_3_CN.

SYML1-Fe: 0.10 g (0.24 mmol) of SYML was dissolved in 20 mL of CH_3_CN. A total of 67 μL (0.48 mmol) of Et_3_N. A pinch of ascorbic acid and 47.73 mg of FeCl_2_·4H_2_O (0.24 mmol) were added to the ligand solution. The resulting solution was stirred for 1 h. A dark brown precipitate was filtered off and then dried with diethyl ether. The precipitate was dissolved in CHCl_3_ and recrystallized by slow difusion with hexane. Dark red crystals were obtained after four days. Yield: 63% (72.6 mg). IR (KBr, cm^−1^): 1617 (s), 1613 (s), 1573 (s), 1530 (s), 1456 (m), 1395 (m), 1360 (s), 1187 (m), 826 (w), 739 (m). MALDI-TOF: [Fe(C_28_H_18_N_2_O_2_)] MW = 470.0 g/mol, Ms/z (M+1H^+^) = 470.0 (M+Na^+^) = 540.0.

SYML2–Mn: 100 mg (0.287 mmol) of four were dissolved in 15 mL of MeOH under conditions of agitation and heat. The resulting solution was red/orange. A total of 113.68 mg (0.574 mmol) of MnCl_2_ · 4H_2_O were added to the previous solution and a color change to dark brown is observed. Furthermore, 80 μL (0.574 mmol) of Et_3_N were added to the reaction mixture to deprotonate the ligand. The solution was heated and stirred for 5 minutes. Slow evaporation of the reaction mixture resulted in a dark brown solid. Yield: 80%.

IR (ATR, cm^−1^): 1607, 1578 (w), 1539(w), 1440 (m), 1248 (s), 1191 (m), 734 (m), 588 (m). LRMS (ESI): (C_46_H_50_N_4_O_14_-Mn_4_) MW = 1102 g/mol it is not observed. (C_20_H_14_N_2_O_4_-Mn) Ms/z (M+1H^+^) = 401.04.

ASYML–Cu: 83.5 mg (0.219 mmol) of ligand ASYML were dissolved in 5 mL of MeOH under conditions of agitation and heat. The resulting solution was orange. 28.37 mg (0.219 mmol) of CuCl_2_ · 2H_2_O were added to the previous solution. A color change from orange to dark red was observed. Then, 61 μL (0.437mmol) of Et_3_N were added. The brown solid obtained was filtered and then dissolved in CH_3_CN for recrystallize by liquid-liquid slow diffusion with hexane. Yield assuming 1 ASYML: 1 Cu molecular formula: 5%. The crystals obtained were too small to obtain good single-crystal X-ray diffraction data. A low-resolution structure shows the chemical composition [Cu(ASYMLH)] for ASYML-Cu.

IR (ATR, cm^−1^): 1614 (w), 1600 (m), 1575 (m), 1535 (m), 1444 (m), 1397 (Cu(ASYML)w), 1364 (s), 1285 (m), 1221 (w), 1193 (s), 1037 (w), 824 (w), 732, 720 (s). MS (ESI): (C_24_H_16_N_2_O_3_-Cu) M+W = 443.64 g/mol Ms/z (M+1H^+^) = 444.06. (M+Na^+^) = 467.06. (2M+H^+^) = 889.10

### Characterization Methods

Avatar 330 FT–IR Thermo Nicolet 5700 with KBr plates and also using the ATR complement. The intensity of the signals is indicated as: (s) strong, (m) medium, and (w) weak. The MS spectra were recorded on the CCiT service. The NMR spectra were recorded on a Varian Mercury 400 MHz, at 298K using CDCl_3_ at the NMR Service Facilities. Coupling constants are given in Hz and the multiplicity of signals is indicated as: (s) singlet, (d) doublet, (t) triplet, and (m) multiplet. CCiT-UB. Mass spectra were collected at the Mass Spectra Service, CCiT-UB. Single-crystal diffraction data for SYML2, SYML1-Cu, SYML1-Fe, ASYML-Cu, and SYML2-Mn were collected on a Bruker APEX II Smart Quazar diffractometer with a Kryoflex low-temperature controller at GMMF facilities. Single-crystal diffraction data for SYML1-Ce were collected on a graphite monochromatic Mo-K. A radiation on a four-circle j geometry KUMA KM-4 diffractometer with a two-dimensional area CCD detector at Polish Academy of Sciences. Magnetic measurements were performed at the Unitat de Mesures Magnètiques of the Universitat de Barcelona on a Quantum Design SQUID MPMS-XL magnetometer equipped with a 5 T magnet. Diamagnetic corrections for the sample holder and for the sample using Pascal’s constants were applied. The treatment of data and the image of the structure was done in the Mercury computer program. The UV–Vis spectra were recorded on a Varian Cary 100 Scan UV–Visible Spectrophotometer. The fluorescence spectra were recorded on an iHR320 Horiba Jobin Yvon imaging spectrometer.

## 4. Conclusions

In this work, we have successfully synthesized and fully characterized a series of new transition metals and cerium complexes from salphen-type Schiff base ligands. The crystal structure of SYML1 revealed that the Schiff base acts as a tetradentate ligand coordinating through N_2_O_2_ and in the presence of Cu(II), Fe(III), and Ce(III) capable of forming different geometries. The reaction of the ligand SYML1 with Cu(II) affords the distorted square planar geometry, as previously reported confirming that a metal that can accommodate coordination number four and is small enough to fit in the N_2_O_2_ cavity will afford mononuclear species. Reactions of SYML1 with Iron or Cerium salts, which are metals that will not accommodate coordination number 4 (Fe) or that are too large for the N_2_O_2_ cavity (Ce) confirm the isolation of polynuclear complex SYML1-Fe for Fe. In this complex, two Fe(III) atoms are distorted as square pyramid geometry and the compound presents an unsupported, nearly linear Fe-O-Fe bridge. The reaction with cerium(III) results in the atypical oxidation of Ce(III) to Ce(IV) to form a monomer, SYML1-Ce, that will be tested as an oxidant in organic media. The reaction with the asymmetric ligand ASYML always result in hydrolysis of the asymmetric ligand to afford the symmetric counterparts and their metal complexes. We have been able to isolate copper in very low yield, which generated a copper mononuclear complex with ASYML, ASYML-Cu. SYML2 is another ligand that has been synthesized and characterized. Our second strategy to obtain polynuclear species adding -OH groups to the salphen backbone leads to the synthesis of the symmetric ligand SYML2. The strategy is successfully followed to obtain a tetranuclear mixed-valent Mn(II)/Mn(III) complex of SYML2, SYML2-Mn. The crystal structure of SYML2-Mn shows that this complex is a tetranuclear complex with two Mn(III) and two Mn(II) in which all of the phenoxo groups of the ligand bridge at least two metal centers. We also report in this paper the magnetic properties of SYML1-Fe, which have a very strong antiferromagnetic interaction between metal centers mediated by the unsupported, nearly linear Fe-O-Fe bridge.

## Figures and Tables

**Table 1:** Crystallographic and data collection parameters for SYML2, SYML1-Ce, SYML1-Fe, SYML1-Cu, and SYML2-Mn, all collected at 100 K.

Compound	SYML2	SYML2-Mn	SYML1-Fe	SYML1-Cu	SYML1-Ce
Formula	C_20_H_16_N_2_O_4_·EtOH	C_46_H_42_Mn_4_N_4_O_14_, 4MeOH	C_56_H_36_N_4_O_5_Fe_2_ 2CHCl_3_	C_28_H_18_CuN_2_O_2_ CH_2_Cl_2_	C_56_H_38_CeN_4_O_5_
Crystal system	monoclinic	triclinic	monoclinic	monoclinic	orthorhombic
Space group	*P 2_1_/n*	*P* $\bar{1}$	*C 2/c*	*P 2_1_/n*	*P2_1_2_1_2*
*a* [Å]	6.1465 (3)	13.0498 (5)	23.8428(13)	11.2153(16)	7.2925(2)
*b* [Å]	14.3296 (6)	13.4777 (5)	13.3162(8)	25.072(4)	25.5351(6)
*c* [Å]	21.8627 (7)	16.4518 (2)	16.7477(10)	8.0918(12)	12.2582(3)
*α* [º]	90	89.486 (2)	90	90	90
*β* [º]	98.039 (3)	75.109 (2)	110.838(2)	92.767(12)	90
*γ* [º]	90	68.798 (2)	90	90	90
V [Å^3^]	1906.68	2594.06 (17)	4969.51	2272.68	2282.66(10)
*Z*	4	4	4	4	2
Final *R* indexes	R = 0.0719	R = 0.0617	*R_1_* = 0.0321	*R_1_* = 0.0491	*R_1_* = 0.0325
	w*R* = 0.2216	w*R* = 0.2057	w*R_2_* = 0.1092	w*R_2_* = 0.1197	w*R_2_* = 0.0804

## Supplementary Materials

Supplementary materials can be found at https://www.mdpi.com/1422-0067/21/10/3574/s1.

## Abbreviations

MDPI	Multidisciplinary Digital Publishing Institute
DOAJ	Directory of open access journals
