# Supplementary material for: Cathecol and Naphtol Groups in Salphen-Type Schiff Bases for the Preparation of Polynuclear Complexes
Source: Int J Mol Sci. 2020 May 18;21(10):3574. doi: 10.3390/ijms21103574 (PMC7278923; doi:10.3390/ijms21103574)

Supplementary Material for

Cathecol and naphtol groups in salphen-type Schiff bases for the preparation of polynuclear complexes

Samira Gholizadeh Dogaheh,[3] Sara Barbero,[1] Joel Barrientos,[1] Jan Janczak[4], Janet Soleimannejad[3]* and E. Carolina Sañudo [1,2,]*

[1] Secció de Química Inorgànica, Departament de Química Inorgànica i Orgànica, C/Martí i Franqués 1-11, Universitat de Barcelona, 08028, Barcelona, Spain; esanudo@ub.edu
[2] Institut de Nanociència i Nanotecnologia, IN2UB, Universitat de Barcelona, 08028, Barcelona, Spain.
[3] School of Chemistry, College of Science, University of Tehran, P.O. Box 14155-6455, Tehran, Iran
[4] Institute of Low Temperature and Structure Research, Polish Academy of Sciences, P.O. Box 1410, 50-950 Wroclaw, Poland
* Correspondence: ECS esanudo@ub.edu; JS janet_soleimannejad@khayam.ut.ac.ir (F.L.)

Electronic spectra of SYML1-Ce

UV-Vis absorption properties of the free ligand SYML1 and the SYML1-Ce complex in CH_3CN solution at the room temperature are shown in Figure S1. The SYML1 absorption spectrum consists of four bands. The strong bands (λ_{max}: 318 nm, ε: 44387 L mol^{-1}cm^{-1} and λ_{max}: 374nm, ε: 40520 L mol^{-1}cm^{-1}), are arising from SYML allowed π - π* transitions. The much weaker bands in the visible region (λ_{max}: 451 nm, ε: 23497 L mol^{-1} cm^{-1} and λ_{max}: 476nm, ε: 20689 L mol^{-1} cm^{-1}) correspond to forbidden n-π* transitions. Actually, the visible bands are related to the chromophores that will coordinate to the metal, phenol and imine groups. Deprotonation of OH groups and coordination of cerium ion to imine and phenoxide groups clearly was happened. The electronic spectra of SYML1-Ce show coalescence of the visible bands, which are blue-shifted with respect to the electronic spectrum of free SYML1. However, the 370 nm band of complexes is red shifted related to free ligand. The bands observed λ_{max}: 380nm, ε: 562200 L mol^{-1} cm^{-1} and λ_{max}: 448nm, ε: 359300 L mol^{-1} cm^{-1} for SYML1-Ce.

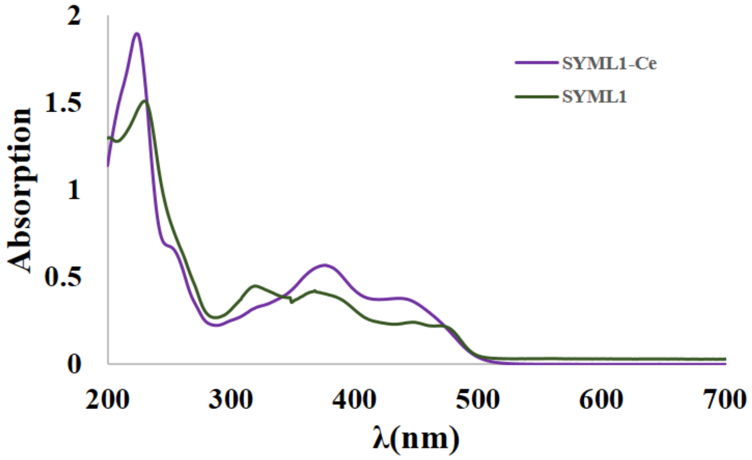

Figure S1. Electronic spectra of **SYML1** and **SYML1-Ce** complex at room temperature.

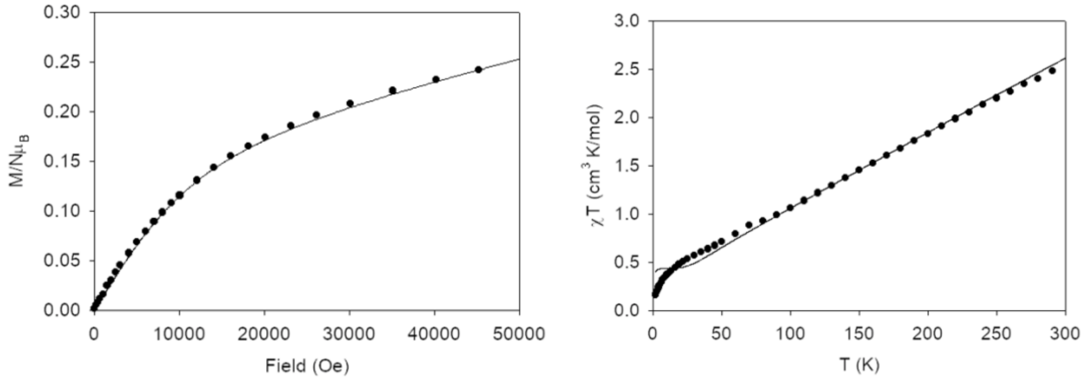

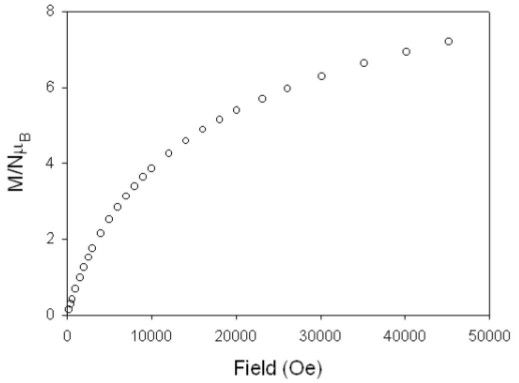

Figure S2. Fitting of the magnetic susceptibility of **SYML1-Fe** with g1=g2 = 2.20, J = -47.85 +/- 0.49 cm^{-1} , TIP = 100e-6 cm^3/mol and 9% paramagnetic impurity of S = 5/2. The fitting has been obtained with PHI (N. F. Chilton, R. P. Anderson, L. D. Turner, A. Soncini and K. S. Murray *J. Comput. Chem.* 34, 1164-1175 (2013). The magnetization vs. field data at 2 K can be fitted with values of J between -120 and -50 cm^{-1} and 3% paramagnetic impurity. All fittings are similar to the one shown with J = -57 cm^{-1}.

Magnetization vs. field plot for **SYML2-Mn** (bottom) at 2 K.

Bond valence sum for the complexes, calculated according to:

I. D. Brown and D. Aletrmatt, *Acta Cryst.*, **1985**, B41, 244-247

I. D. Brown and Kang Kun Wu ,*Acta Cryst.* **1976**. B32, 1957

Wentian Liu and H. Holden Thorp , *Inorg. Chem.* **1993**, 32, 4102-4105

Gus J. Palenik, Sheng-Zhi Hu, *Inorganica Chimica Acta* , **2009**, *362*, 4740–4743

Table ESI1. Oxidation state of the metals at the complexes

Complex	Metal	BVS Cu(I)	BVS Cu(II)
ASYML-Cu	Cu	–	**2.40**
**Complex**	**Metal**	**BVS Fe(II)**	**BVS Fe(III)**
SYML1-Fe	Fe	2.75	**3.26**
**Complex**	**Metal**	**BVS Ce(III)**	**BVS Ce(IV)**

SYML1-Ce	Ce1	4.03	**3.94**
**Complex**	**Metal**	**BVS Mn(II)**	**BVS Mn(III)**
SYML2-Mn	Mn1	3.42	**3.16**
	Mn2	3.43	**3.17**
	Mn3	**2.28**	2.13
	Mn4	**2.29**	2.15

Table ESI2. Protonation state of the ligands at the complexes, only calculated for those complexes with ambiguous protonation.

Complex		Oxygen	BVS-	
ASYML-Cu	ASYMLH ligand	O1	1.82	deprotonated
		O2	1.82	deprotonated
		O3	0.99	protonated
SYML1-Fe	Oxo bridge	O1	2.08	oxo bridge
SYML2-Mn	SYML2 ligand	O1	1.91	deprotonated
		O2	1.90	deprotonated
		O5	1.88	deprotonated
		O6	1.90	deprotonated
		O3	1.67	deprotonated
		O4	1.68	deprotonated
		O7	1.69	deprotonated
		O8	1.71	deprotonated
	Terminal MeOH	O11	1.13	protonated
		O12	1.12	protonate
		O13	1.14	protonate
		O14	1.16	protonate
	MeO- bridges	O9	2.06	deprotonated
		O10	2.07	deprotonated

Table ESI3: Crystallographic parameters for **ASYMLH-Cu.**

**Compound**	**ASYMLH-Cu**
Formula	C24 H16 Cu N2 O3, H2 O [+ solvent]
Crystal system	monoclinic
Space group	$P\ 2_1/c$
a [Å]	22.691(15)
b [Å]	21.074(12)
c [Å]	8.332(5)
α [º]	90
β [º]	90.28
γ [º]	90
V [Å^3]	3984(4)
Z	8
Final R indexes	R=0.0890
	wR = 0.1705

Figure S3. Mass spectra of the metal complexes:

SYML1-Cu:

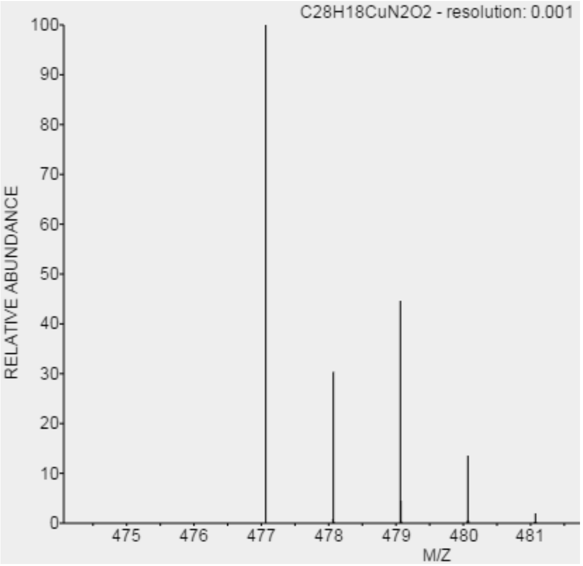

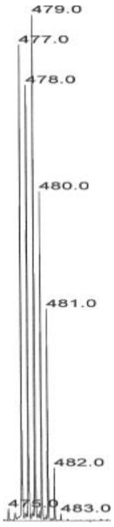

SYML1-Fe:

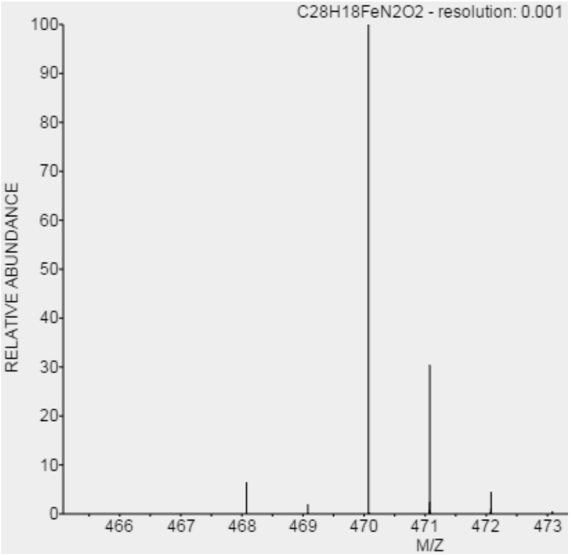

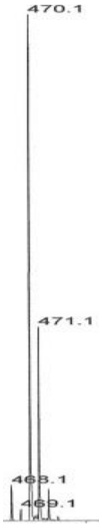

SYML2-Mn:

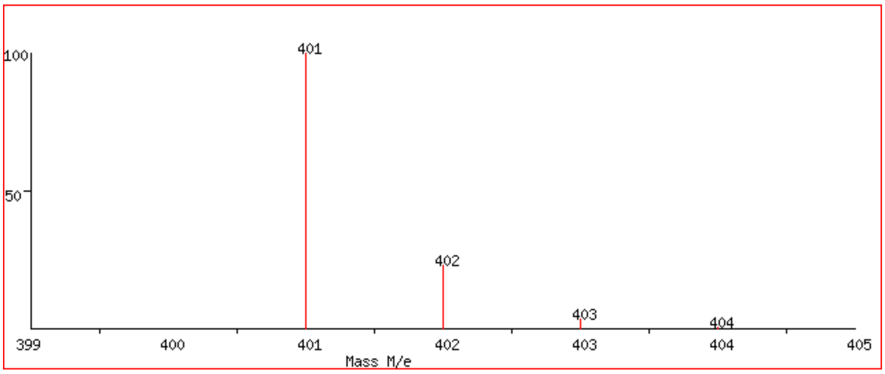

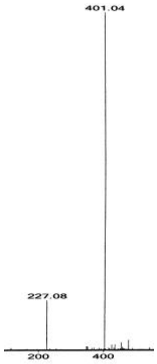

ASYML-Cu:

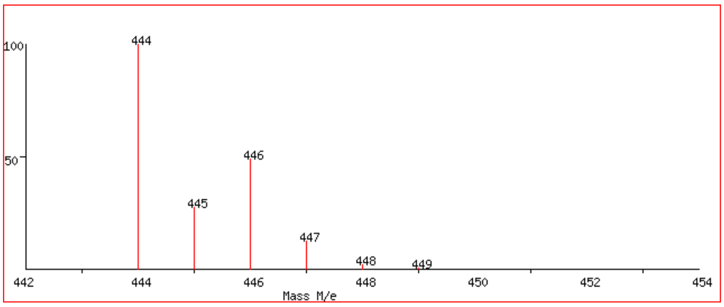

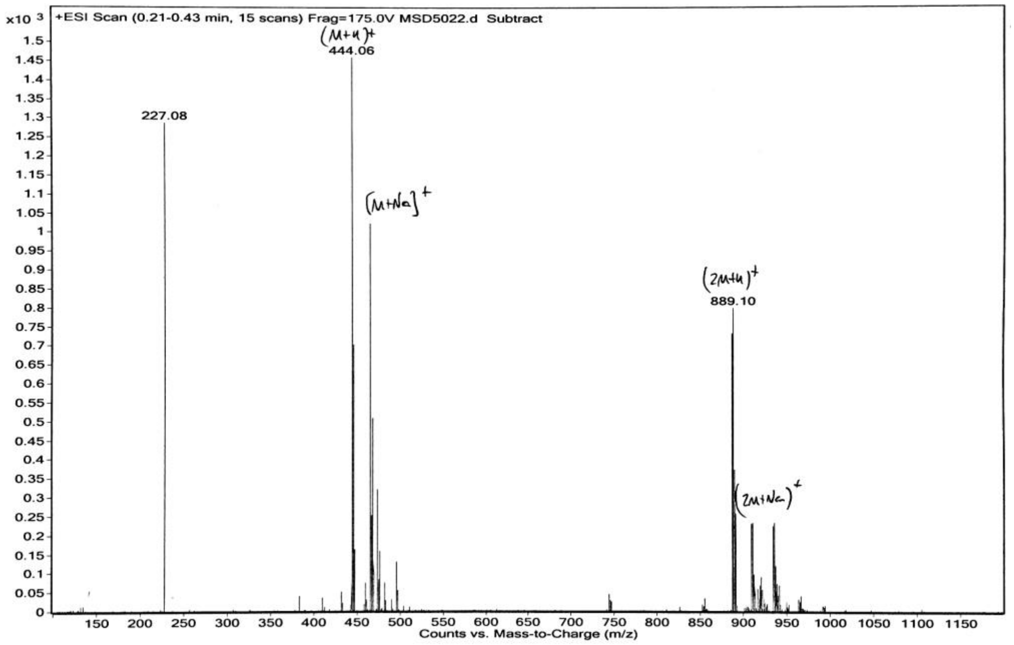

Figure S4. Proton NMR in $CDCl_3$ and ESI MS of ASYML obtained by the one-pot synthesis method.

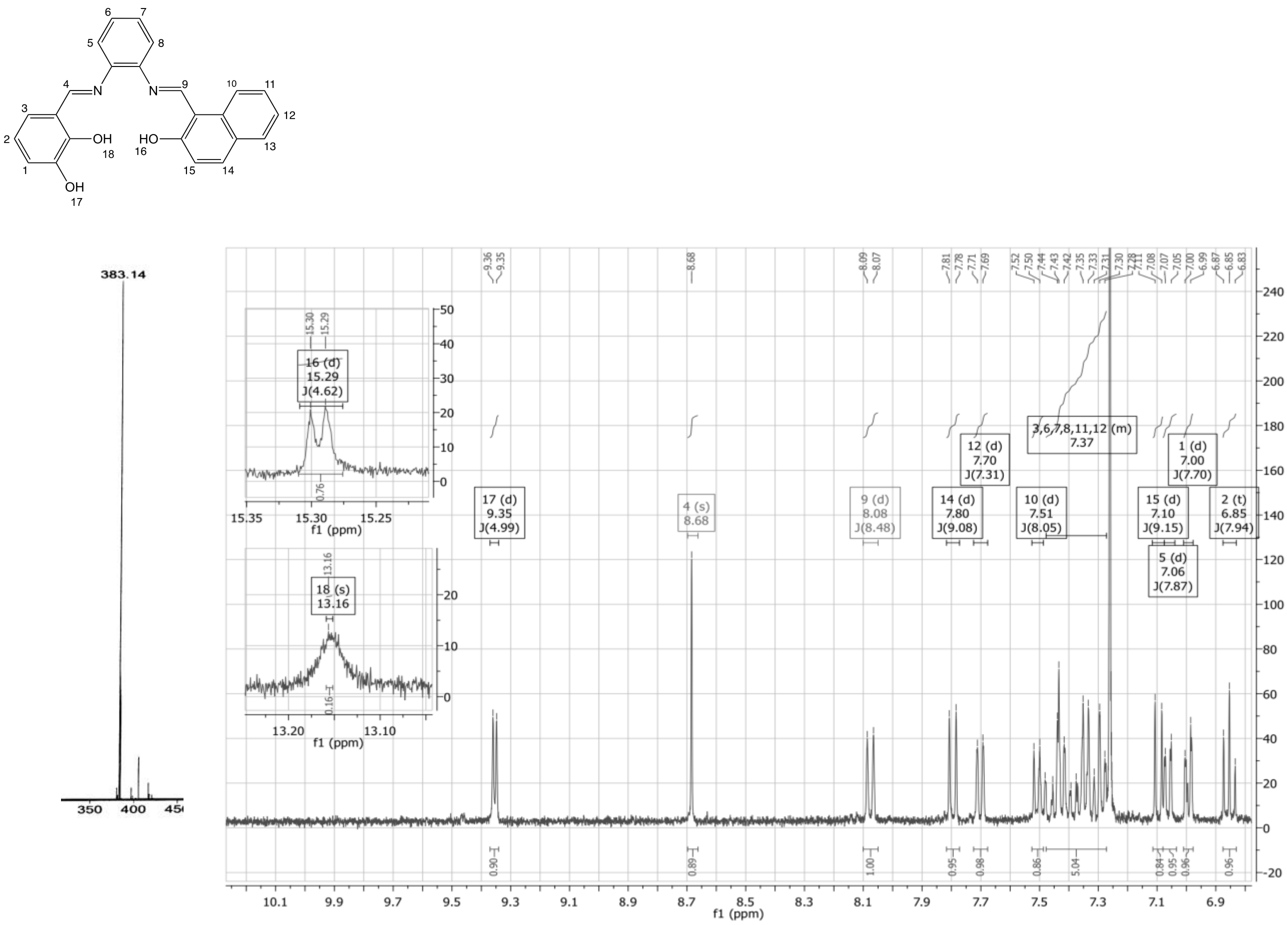

Supplement: Supplementary file 1 [file ijms-21-03574-s001.pdf]
